# AI-enabled instructor care and physical fitness performance in police cadets: the mediating roles of emotions, engagement, and recovery

**DOI:** 10.3389/fpsyg.2026.1800027

**Published:** 2026-07-09

**Authors:** Zeyu Zhang, Mahpiret Kanji, Xiaomei Lu

**Affiliations:** 1 Judicial Police College, Xinjiang University of Political Science and Law, Urumqi, China; 2 School of Educational Science, Xinjiang Normal University, Urumqi, China; 3 Xinjiang Key Laboratory of Mental Development and Learning Science, Xinjiang Normal University, Urumqi, China; 4 Hospital of Sihong, Suqian, China

**Keywords:** affective processes, AI-supported care, burnout, physical fitness performance, police training, sleep quality, training engagement

## Abstract

**Background:**

Police training is physically demanding and psychologically stressful, which may undermine training adaptation and physical performance. Artificial intelligence (AI)-supported learning analytics systems have been proposed as scalable tools to support training management, yet empirical evidence remains limited regarding how such systems may operate when embedded in human instructor care practices.

**Methods:**

We conducted a quasi-experimental pre-post study among police cadets (*N* = 224) from four natural classes in a second-level police college. Two classes (*n* = 112) received an AI-enabled instructor care dashboard intervention, while two classes (*n* = 112) followed routine training. The dashboard generated weekly care signals based on non-sensitive training and self-report indicators and prompted instructors to provide standardized supportive actions, including brief conversations and individualized training suggestions. Physical fitness performance (0–100) was assessed at the beginning (T0) and end (T1) of the semester. Difference-in-differences (DID) models with covariate adjustment and class fixed effects were used to estimate intervention effects. Exploratory mediation analyses examined whether post-intervention emotional states, training engagement, sleep quality, and burnout were associated with the observed intervention effect.

**Results:**

DID estimates indicated a significant net improvement in physical fitness performance in the AI-enabled instructor care condition (*β* = 3.48, *p* < 0.001). Exploratory mediation analyses suggested that this improvement was statistically associated with more positive emotional states, higher training engagement, better sleep quality, and lower burnout.

**Conclusion:**

AI-supported instructor care may represent a low-risk and scalable approach to supporting physical training performance in high-stress educational settings. The findings provide quasi-experimental evidence consistent with an intervention effect, while the mediation results should be interpreted as exploratory evidence of associated psychological and recovery-related pathways.

## Introduction

Police training requires sustained physical exertion under conditions of high psychological stress. Cadets are frequently exposed to intensive training schedules, strict disciplinary systems, and continuous performance evaluation, all of which place substantial demands on both physical and emotional resources. Empirical studies in police and military populations consistently indicate that chronic stress and maladaptive psychological states—such as negative affect, burnout, and sleep disturbances—are associated with impaired recovery, reduced training effectiveness, and elevated injury risk (Violanti et al., 2018; Garbarino et al., 2013; Arnetz et al., 2009). In this context, physical performance cannot be understood solely as a function of training load, but must also be examined in relation to cadets’ emotional regulation and recovery processes.

Recent advances in learning analytics and artificial intelligence (AI) provide new opportunities to support training processes by detecting behavioral risk signals and enabling timely interventions. AI-enabled systems have demonstrated potential in improving student engagement, persistence, and academic outcomes across diverse educational settings (Siemens and Long, 2011; Viberg et al., 2018; Holmes et al., 2019). However, most existing applications of AI in education emphasize automated feedback or outcome prediction, with limited attention to how AI-supported systems interact with human care practices. In high-risk training environments such as police education, purely automated feedback may be insufficient or even counterproductive, as cadets often require emotionally sensitive, context-aware support. Therefore, AI systems that function as decision-support tools for instructors—rather than as autonomous intervention agents—may represent a more theoretically grounded and ethically viable approach.

**Figure 1:**
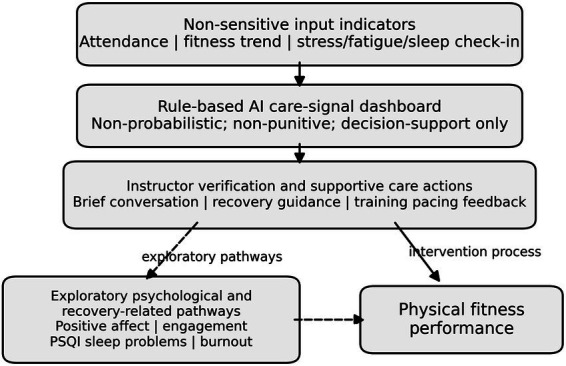
Conceptual framework of the bundled AI-enabled instructor care model. The model presents the AI dashboard as a rule-based decision-support tool that generates care signals from non-sensitive training and self-report indicators. These signals prompt instructor verification and supportive care actions. Emotional states, training engagement, PSQI sleep problems, and burnout are presented as exploratory psychological and recovery-related pathways rather than definitive causal mediators.

From a psychological perspective, care-oriented interventions are expected to influence performance through multiple interrelated processes. First, perceived care from instructors may improve emotional states by reducing anxiety and enhancing positive affect, consistent with social support and self-determination theories (Reeve, 2012; Ryan and Deci, 2017). Second, emotional improvement is likely to foster higher engagement, reflected in greater persistence, dedication, and training commitment (Hagger and Chatzisarantis, 2009; Schaufeli et al., 2006). Third, supportive interactions may facilitate recovery by improving sleep quality and reducing burnout, which are critical determinants of sustained physical performance in high-demand occupations (Sonnentag and Fritz, 2015; McCraty and Atkinson, 2012). Together, these processes suggest a psychological pathway linking care-oriented interventions to objective performance outcomes via emotions, engagement, and recovery processes (Figure 1). Conceptually, this framework is consistent with integrative stress-recovery models in occupational psychology, which emphasize the joint role of affective regulation, motivational engagement, and recovery processes in sustaining performance under prolonged stress.

Despite growing interest in AI-assisted training systems, empirical evidence on their psychological and recovery-related pathways remains scarce, particularly in public safety education. Most existing studies rely on correlational designs or focus on academic outcomes, offering limited insight into intervention effects on physical training performance. Moreover, few studies employ quasi-experimental designs with objective institutional indicators as outcome measures. To address these gaps, the present study examines whether an AI-enabled instructor care dashboard is associated with greater improvement in physical fitness performance among police cadets and explores emotional states, engagement, sleep quality, and burnout as potential mechanistic correlates. By integrating learning analytics with psychologically grounded human care practices, this study provides quasi-experimental evidence consistent with a scalable, low-risk intervention model in physically intensive training contexts.

## Methods

### Participants and design

A quasi-experimental pre-post design with nonequivalent groups was employed. Participants were 224 police cadets recruited from four natural classes within the same grade of a second-level police training institution in Western China. Two classes (*n* = 112) were assigned to the intervention group, and two classes (*n* = 112) served as the control group. Group assignment was conducted at the class level to ensure feasibility and minimize contamination across participants.

All participants completed assessments at the beginning of the semester (T0) and at the end of the semester (T1). The study protocol was approved by the institutional ethics committee, and informed consent was obtained from all participants prior to data collection. Participation was voluntary, and the use of dashboard-generated signals was non-punitive and did not affect academic grades, disciplinary evaluation, or training certification.

### Intervention: AI-enabled instructor care dashboard

The intervention consisted of an AI-enabled instructor care dashboard (AID-CS) designed to generate weekly care signals based on non-sensitive training and self-report indicators. Input signals included training attendance records, weekly training performance trends, and brief self-reported measures of stress, fatigue, and sleep quality (three-item weekly check-in).

In the present study, the term “AI-enabled” refers to a rule-based learning analytics and decision-support dashboard that automatically integrated multiple weekly indicators, detected deviations from individual baseline patterns, and generated care prompts for instructors. The system did not involve adaptive machine-learning model training or autonomous decision-making.

The AID-CS dashboard used a rule-based, non-probabilistic signal detection procedure rather than a black-box machine learning model. The system was designed as a decision-support tool for instructors and did not make autonomous training, disciplinary, or evaluative decisions. Weekly input indicators included training attendance, weekly physical training performance trends, and a brief three-item self-report check-in assessing perceived stress, fatigue, and sleep quality.

For each cadet, weekly indicators were compared with the individual’s own baseline and recent trend. A care signal was generated when one or more indicators showed a meaningful negative deviation from the cadet’s usual pattern, such as repeated absence, a decline in weekly performance, elevated fatigue or stress, or poorer sleep quality. Based on the number and persistence of deviation signals, cadets were classified into three care levels: normal, attention, and high-risk. Normal indicated no obvious deviation signal; attention indicated one or two temporary deviation signals requiring routine instructor awareness; and high-risk indicated repeated or combined deviation signals requiring timely supportive follow-up.

In operational terms, an attention-level signal was generated when one indicator showed a negative deviation for two consecutive weeks or when two indicators showed concurrent negative deviations within the same week. A high-risk signal was generated when three or more indicators showed concurrent negative deviations or when an attention-level signal persisted for three consecutive weeks. These thresholds were used as practical care-trigger rules rather than diagnostic cutoffs. Because the dashboard was developed for internal training support, some institution-specific weighting details cannot be fully disclosed; however, the above rules describe the core classification logic used in the present study.

The same decision rules were used throughout the 8-week intervention period and were not updated adaptively during the study. To reduce the risk of false positives and false negatives, dashboard signals were not treated as diagnostic labels. Instead, each signal functioned as a prompt for instructors to verify the cadet’s situation through brief supportive conversations. No punitive action, grade penalty, or administrative consequence was triggered by the dashboard. Instructor responses were limited to standardized care actions, including short one-on-one conversations, recovery guidance, training pacing suggestions, and motivational feedback.

Instructors conducted standardized human care actions following dashboard prompts, including brief one-on-one conversations and individualized training adjustment suggestions. The intervention was implemented once per week over an 8-week period. The control group followed routine training procedures without access to the care dashboard or structured weekly instructor feedback.

## Measures

### Physical fitness performance

Physical fitness performance was operationalized using the official institutional composite fitness score (range 0–100), derived from standardized physical assessments conducted by the training institution. The composite score was obtained at both T0 and T1 and served as the primary outcome variable.

### Emotional states

Positive and negative affect were assessed using the International Positive and Negative Affect Schedule Short Form (I-PANAS-SF), which consists of 10 items (5 for positive affect and 5 for negative affect). Participants rated each item on a 5-point Likert scale (1 = very slightly or not at all, 5 = extremely). Internal consistency in the present sample was satisfactory (Cronbach’s *α* = 0.86 for PA and 0.84 for NA). The primary mediation model focused on positive affect because the conceptual framework emphasized adaptive emotional resources; negative affect was assessed to describe emotional states but was not specified as a focal mediator in the main model.

### Training engagement

Training engagement was measured using the Utrecht Work Engagement Scale (UWES-9). The scale contains 9 items rated on a 5-point Likert scale (1 = never, 5 = always), assessing vigor, dedication, and absorption. The overall engagement score was computed as the mean of all items (Cronbach’s *α* = 0.91).

### Sleep quality

Sleep quality was assessed using the Pittsburgh Sleep Quality Index (PSQI). The PSQI yields a global score ranging from 0 to 21, with higher scores indicating poorer sleep quality and lower scores indicating better sleep quality. Internal consistency was acceptable (Cronbach’s *α* = 0.79).

### Burnout

Burnout was measured using the Maslach Burnout Inventory-Student Survey (MBI-SS). The scale includes three dimensions: emotional exhaustion, cynicism, and reduced professional efficacy. Items were rated on a 5-point Likert scale, and the overall burnout score was computed as the mean of all items (Cronbach’s *α* = 0.88).

### Statistical analysis

Intervention effects on physical fitness performance were estimated using difference-in-differences (DID) regression models:


$$\begin{array}{c} {\text{Fitness}}_{\mathrm{it}}=\beta_{0}+\beta_{1}{\text{Treat}}_{i}+\beta_{2}{\text{Post}}_{t}+\beta_{3}({\text{Treat}}_{i}\times {\text{Post}}_{t})\\ +\gamma X_{i}+\delta_{\text{class}}+\varepsilon_{\mathrm{it}} \end{array}$$

Where Fitness*_it_* represents physical fitness score for individual *i* at time *t*, Treat*_i_* indicates group assignment (1 = intervention, 0 = control), and Post*_t_* indicates post-test (T1). *X_i_* includes covariates (sex, age, baseline fitness), and *δ*_class_ denotes class fixed effects. Robust standard errors were used. The interaction term Treat × Post was interpreted as the estimated intervention effect.

Because the study included only two waves of data, the parallel trends assumption underlying DID could not be formally tested. Baseline equivalence between the intervention and control groups, covariate adjustment, and class fixed effects were used to reduce potential confounding, but these procedures cannot fully eliminate the possibility of unobserved class-level differences. Therefore, DID estimates are interpreted as quasi-experimental evidence consistent with an intervention effect rather than as definitive causal proof.

To examine potential psychological and recovery-related pathways, exploratory parallel multiple mediation analyses were conducted using bootstrapping (5,000 resamples) with positive affect, engagement, sleep quality, and burnout as mediators. Because mediators and the physical fitness outcome were assessed within the same post-treatment period, these analyses were interpreted as exploratory evidence of associated mechanistic correlates rather than strict causal mediation.

As a descriptive robustness check, we compared the pre-post change scores in the two groups. This change-score comparison was used to examine whether the direction and magnitude of the mean difference were consistent with the DID estimate.

## Results

### Descriptive statistics and baseline comparability

Descriptive statistics for all key variables are presented in Table 1. At baseline (T0), no significant differences were observed between the intervention and control groups in physical fitness performance, positive affect, training engagement, sleep quality, or burnout (all *p* > 0.10), indicating satisfactory group comparability prior to the intervention.

**Table 1:** Means and standard deviations of key variables at baseline (T0) and post-test (T1).

Variable	Group	T0 mean (SD)	T1 mean (SD)
Physical fitness (0–100)	Control	72.1 (8.3)	73.3 (8.1)
	Intervention	71.8 (8.5)	76.5 (8.0)
Positive affect (1–5)	Control	3.28 (0.58)	3.31 (0.56)
	Intervention	3.26 (0.60)	3.63 (0.55)
Engagement (1–5)	Control	3.14 (0.55)	3.19 (0.52)
	Intervention	3.12 (0.56)	3.46 (0.50)
PSQI (0–21)	Control	7.6 (3.0)	7.3 (2.9)
	Intervention	7.7 (3.1)	6.2 (2.8)
Burnout (1–5)	Control	2.91 (0.57)	2.86 (0.56)
	Intervention	2.92 (0.58)	2.62 (0.55)

No significant baseline differences were found between groups (all *p* > 0.10).

At post-test (T1), the intervention group showed higher levels of physical fitness, positive affect, and engagement, as well as lower levels of sleep problems and burnout, compared to the control group. Because higher PSQI scores indicate poorer sleep quality, the decrease in PSQI scores in the intervention group should be interpreted as an improvement in sleep quality. Notably, the mean physical fitness score of the intervention group increased from 71.8 (SD = 8.5) at T0 to 76.5 (SD = 8.0) at T1, whereas the control group exhibited only a modest increase from 72.1 (SD = 8.3) to 73.3 (SD = 8.1).

### Intervention effects on physical fitness performance

To estimate the intervention effect of the AI-enabled instructor care dashboard on physical fitness performance, difference-in-differences (DID) regression models were conducted. Regression results are presented in Table 2.

**Table 2:** Difference-in-differences regression results predicting physical fitness performance.

Predictor	*β*	SE	*t*	*p*
Treat	−0.31	0.67	−0.46	0.646
Post	1.12	0.51	2.20	0.029
Treat × post	3.48	0.82	4.24	<0.001
Sex	1.95	0.58	3.36	0.001
Age	−0.14	0.09	−1.56	0.120
Baseline fitness	0.61	0.05	12.20	<0.001

Models include class fixed effects. Robust standard errors are reported.

The interaction term between group and time (Treat × Post) was positive and statistically significant (*β* = 3.48, SE = 0.82, *p* < 0.001), indicating that cadets in the intervention group achieved an average net improvement of 3.48 points in physical fitness performance relative to the control group after adjusting for sex, age, baseline fitness, and class fixed effects. This result provides quasi-experimental evidence consistent with a positive intervention effect associated with the AI-supported instructor care model.

Among control variables, baseline fitness emerged as a strong predictor of post-test performance (*β* = 0.61, *p* < 0.001), suggesting substantial stability in individual fitness levels across the training period. Sex was also significantly associated with physical fitness outcomes (*β* = 1.95, *p* = 0.001), whereas age was not significantly related to performance (*β* = −0.14, *p* = 0.120).

The estimated net improvement of 3.48 points on the 0–100 institutional fitness scale may be practically meaningful in police training contexts. Relative to the baseline standard deviation of physical fitness scores, this effect represents a modest but non-trivial improvement. In structured police training, even small gains in composite fitness performance may influence readiness evaluation, training classification, and the identification of cadets who require additional support. The descriptive change-score comparison was also consistent with the DID estimate: the intervention group increased by 4.7 points from T0 to T1, whereas the control group increased by 1.2 points, yielding a descriptive net difference of 3.5 points.

**Figure 2:**
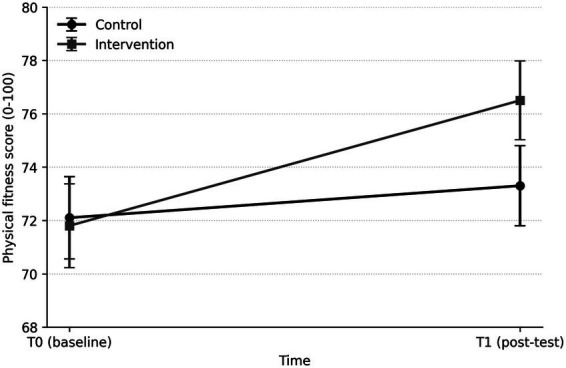
Pre–post changes in physical fitness performance by study condition. Values represent raw mean physical fitness scores at baseline (T0) and post-test (T1). Error bars indicate 95% confidence intervals. The difference-in-differences estimate is reported in Table 2.

A visual depiction of pre-post changes in physical fitness for both groups is shown in Figure 2. The two-line trajectory highlights the similar baseline scores and the larger improvement from T0 to T1 in the AI-enabled instructor care condition.

### Exploratory mediation analysis: psychological and recovery-related pathways

To examine psychological and recovery-related pathways associated with the intervention effect, exploratory parallel multiple mediation analyses were conducted with positive affect, training engagement, PSQI sleep-problem scores, and burnout entered simultaneously as mediators. Bootstrapping with 5,000 resamples was applied to estimate indirect effects and corresponding 95% bias-corrected confidence intervals. Mediation analyses were conducted using a regression-based approach equivalent to PROCESS Model 4. Because mediators and the physical fitness outcome were assessed within the same post-treatment period, the results should be interpreted as evidence of associated mechanistic correlates rather than definitive causal mediation.

As reported in Table 3, the total indirect effect of the intervention on physical fitness performance was statistically significant [*β* = 1.62, 95% CI (0.88, 2.49)], suggesting that post-intervention psychological and recovery-related indicators were statistically associated with the observed intervention effect. Specifically, positive affect exhibited an indirect effect of *β* = 0.52 [95% CI (0.22, 0.95)], training engagement showed an indirect effect of *β* = 0.41 [95% CI (0.14, 0.78)], PSQI sleep-problem scores demonstrated an indirect effect of *β* = 0.43 [95% CI (0.16, 0.89)], and burnout yielded an indirect effect of *β* = 0.26 [95% CI (0.07, 0.58)]. Because higher PSQI scores indicate poorer sleep quality, the sleep-related pathway should be interpreted as involving lower sleep problems and better recovery.

**Table 3:** Indirect effects of AI-enabled instructor care on physical fitness performance.

Mediator	Indirect effect	95% CI
Positive affect	0.52	[0.22, 0.95]
Engagement	0.41	[0.14, 0.78]
PSQI sleep problems	0.43	[0.16, 0.89]
Burnout	0.26	[0.07, 0.58]

Results are based on exploratory parallel multiple mediation analyses using bootstrapping with 5,000 resamples. Values represent standardized indirect effects with 95% bias-corrected confidence intervals. Positive affect, training engagement, PSQI sleep-problem scores, and burnout were included simultaneously as mediators. Higher PSQI scores indicate poorer sleep quality; therefore, the sleep-related pathway should be interpreted as involving lower sleep problems and better recovery. Confidence intervals that do not include zero indicate statistically significant indirect effects.

Importantly, the direct effect of the intervention remained statistically significant after accounting for all mediators [*β* = 1.86, 95% CI (0.62, 3.15)], indicating a pattern of partial mediation. This result suggests that psychological and recovery-related correlates are associated with part of the intervention effect, while additional unmeasured processes may also contribute to performance improvement.

**Figure 3:**
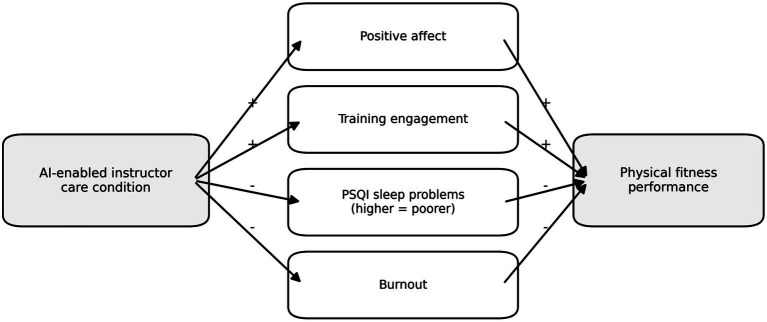
Exploratory mediation model linking AI-enabled instructor care to physical fitness performance. Signs indicate the direction of associations. PSQI sleep-problem scores were used to represent sleep quality, with higher scores indicating poorer sleep quality. The mediation model is exploratory because mediators and physical fitness performance were assessed within the same post-treatment period. The direct effect of the intervention remained significant after accounting for the mediators.

A graphical representation of the exploratory mediation model is presented in Figure 3, which illustrates the associated pathways linking AI-enabled instructor care to physical fitness performance via emotional states, training engagement, PSQI sleep-problem scores, and burnout. The previous descriptive bivariate sleep-quality plot was removed to avoid overinterpreting a post-test correlation as evidence of causal mediation.

## Discussion

### Principal findings

The present study provides quasi-experimental evidence consistent with an intervention effect of an AI-enabled instructor care dashboard on physical fitness performance among police cadets in a high-stress training context. Results from the DID analysis indicated that cadets receiving AI-supported instructor care achieved significantly greater improvements in physical fitness compared with those undergoing routine training alone. Importantly, this association remained robust after controlling for demographic factors, baseline fitness, and class-level fixed effects. These findings suggest that integrating AI-generated care signals with human instructor actions may support objective physical training outcomes in high-demand educational contexts.

### Interpretation of the findings: psychological and recovery-related pathways

From a psychological perspective, the exploratory mediation analyses suggest that the intervention effect was statistically associated with emotional, motivational, and recovery-related indicators. Cadets in the AI-enabled instructor care condition reported more positive emotional states, higher training engagement, better sleep quality, and lower burnout, and these post-intervention indicators were associated with better physical fitness performance. However, because mediators and outcomes were assessed within the same post-treatment period, these findings should be interpreted as evidence of associated mechanistic correlates rather than definitive causal mediation.

Rather than directly influencing performance, AI systems appear to operate indirectly by enabling instructors to deliver timely, context-sensitive human care, thereby activating psychological mechanisms that support sustained physical adaptation. From a human factors perspective, this indirect pathway is particularly relevant in high-stress training environments, where emotional load and sustained attention are critical determinants of performance outcomes Hancock and Warm (1989). By reducing maladaptive stress responses and facilitating recovery, instructor-mediated care may help stabilize performance trajectories under prolonged physical and psychological demands.

### Theoretical implications for human-centered AI and organizational psychology

From a human factors and organizational psychology perspective, this study advances existing models of AI-enabled interventions by demonstrating how human-centered, decision-support systems can enhance performance through relational and organizational processes. From a human factors perspective, this design is consistent with classical models of human-automation interaction, which emphasize that automated systems are most effective when they support human judgment and decision-making rather than fully replacing it (Parasuraman et al., 2000; Shneiderman, 2020).

Previous research has predominantly conceptualized AI as a tool for prediction, monitoring, or individualized recommendation (Siemens and Long, 2011; Viberg et al., 2018). In contrast, our findings highlight the importance of positioning AI as a decision-support system that enhances instructors’ capacity for human care rather than replacing it. This design logic aligns closely with principles of human-centered artificial intelligence, which emphasize that AI systems should augment professional judgment, preserve human agency, and support reliable performance under conditions of cognitive and emotional strain (Shneiderman, 2020).

Particularly in physically intensive and high-risk training environments, such as police education, emotionally sensitive and relational support may be more impactful than purely algorithmic feedback. This perspective aligns with recent calls for responsible and human-in-the-loop AI in educational settings (Holmes et al., 2019). Theoretically, this study extends existing models of AI in education by conceptualizing AI not as a primary intervention agent, but as a socio-technical trigger that activates human relational mechanisms within organizational contexts.

### Practical implications for high-stress training contexts

The present findings suggest that AI-enabled care dashboards can serve as scalable and ethically responsible support tools for enhancing performance and well-being in high-stress training environments. First, AI-enabled care dashboards may support training outcomes without increasing training load or relying solely on specialized psychological personnel. Second, by emphasizing instructor-mediated care actions, this model avoids ethical concerns associated with automated risk labeling and preserves the central role of human judgment in training decisions. The estimated 3.48-point net improvement in physical fitness performance is modest but practically meaningful in a police training context, where composite fitness scores are often used to evaluate readiness and identify cadets requiring additional support.

From an organizational psychology perspective, instructor care can also be interpreted as a form of perceived organizational support, which has been shown to buffer stress, enhance engagement, and promote adaptive performance in demanding work contexts (Eisenberger et al., 2016). For public safety institutions, adopting AI-supported care systems may therefore offer an evidence-based pathway to enhance training effectiveness while safeguarding cadets’ psychological well-being.

### Limitations and directions for future research

Several limitations should be considered when interpreting the present findings, which also point to important directions for future research. First, group assignment was conducted at the class level rather than through individual randomization. Although this approach was necessary to reduce contamination across cadets and to preserve the integrity of the training organization, it may introduce selection bias and unobserved class-level confounding. Factors such as instructor style, class climate, prior instructor-cadet relationships, peer norms, or informal support practices may have influenced the observed outcomes. Baseline equivalence, covariate adjustment, and class fixed effects partially mitigate this concern, but they do not fully rule out alternative explanations. Accordingly, the findings should be interpreted as quasi-experimental evidence consistent with an intervention effect rather than definitive causal proof. In addition, the number of class clusters was small, which limits the precision of cluster-level statistical inference and reduces the extent to which class-level heterogeneity can be fully modeled. The results should therefore be interpreted as evidence from a small-cluster quasi-experimental design rather than as estimates from a fully powered cluster-randomized trial.

Second, the present design evaluated a bundled AI-enabled instructor care model rather than isolating the independent effect of the algorithmic component. The observed effects therefore cannot be attributed solely to the AI dashboard. Instead, they should be understood as the combined result of AI-generated care signals, structured weekly instructor attention, and standardized supportive follow-up actions. Future studies could compare AI-enabled instructor care, non-AI structured instructor care, and routine training conditions to disentangle the unique contribution of AI-generated signals from the effects of increased care frequency and structure.

Third, although the mediation analysis was theoretically grounded, mediators and outcomes were measured within the same post-treatment period. This design does not fully establish temporal precedence for causal mediation. Future studies should collect mediator measures at multiple time points, such as mid-intervention emotional states, engagement, and recovery indicators, followed by later physical performance outcomes. Such designs would allow stronger tests of temporal ordering and mechanism.

Fourth, the intervention was implemented within a single training institution, which may limit the generalizability of the findings to other educational or cultural contexts. Future research should replicate this design across multiple police or public safety training institutions to assess the robustness of the observed effects.

Finally, future studies could explore more fine-grained AI algorithms and adaptive care strategies, such as dynamic risk modeling or personalized feedback thresholds, to optimize the balance between automation and human intervention. Longitudinal designs with extended follow-up periods are also needed to examine the sustainability of AI-supported care effects over time.

## Conclusion

This study provides quasi-experimental evidence consistent with an intervention effect of an AI-enabled instructor care model on physical fitness performance among police cadets. By integrating learning analytics with instructor-mediated care practices, the intervention was associated with greater improvement in physical fitness performance compared with routine training.

The exploratory mediation results further suggest that emotional states, training engagement, sleep quality, and burnout may serve as important psychological and recovery-related correlates of this improvement. However, these pathways should be interpreted cautiously because the mediators and outcome were assessed within the same post-treatment period.

Overall, the findings contribute to human-centered AI and organizational psychology by showing how AI can function as a decision-support tool that prompts timely human care rather than replacing professional judgment. In high-stress training contexts, such socio-technical designs may help support both performance and psychological sustainability, provided that algorithmic signals remain transparent, non-punitive, and embedded in responsible human oversight.

## Data Availability

The raw data supporting the conclusions of this article will be made available by the authors, without undue reservation.
